# Current Landscape and Future Directions in Cancer Immunotherapy: Therapies, Trials, and Challenges

**DOI:** 10.3390/cancers17050821

**Published:** 2025-02-27

**Authors:** Shehani Bandara, Sreejith Raveendran

**Affiliations:** 1 School of Health and Life Sciences, Teesside University, Middlesbrough TS1 3BX, UK; 2 National Horizons Centre, Teesside University, Darlington DL1 1HG, UK

**Keywords:** cancer immunotherapy, clinical trials, nanotechnology, personalised medicine, treatment resistance

## Abstract

Cancer remains a leading global health challenge, placing immense burdens on individuals and healthcare systems. Despite advancements in traditional treatments, significant limitations persist, including treatment resistance, severe side effects, and disease recurrence. Immunotherapy has emerged as a promising alternative, leveraging the immune system to target and eliminate tumour cells. However, challenges such as immunotherapy resistance, patient response variability, and the need for improved biomarkers limit its widespread success. This review provides a comprehensive analysis of the current landscape of cancer immunotherapy, highlighting both FDA-approved therapies and novel approaches in clinical development. It explores immune checkpoint inhibitors, cell and gene therapies, monoclonal antibodies, and nanotechnology-driven strategies, offering insights into their mechanisms, efficacy, and limitations. By integrating emerging research and clinical advancements, this review underscores the need for continued innovation to optimise cancer immunotherapy and overcome existing treatment barriers.

## Simple Summary

Cancer remains a major global health challenge, with existing treatments often facing limitations such as resistance, severe side effects, and disease recurrence. Immunotherapy has revolutionised cancer treatment by leveraging the body’s immune system to recognise and eliminate cancer cells. This review explores various immunotherapeutic approaches, including immune checkpoint inhibitors, monoclonal antibodies, oncolytic viruses, and cell-based therapies, discussing their mechanisms, effectiveness, and challenges. Additionally, emerging technologies such as nanotechnology and gene therapy are being integrated to enhance treatment precision and overcome resistance. While immunotherapy has significantly improved patient outcomes, challenges such as variable patient responses and immune-related side effects persist. Ongoing research focuses on optimising combination therapies and developing personalised treatments to improve efficacy. The findings presented in this review highlight the continuous advancements in cancer immunotherapy, offering new hope for patients and shaping the future of cancer care.

## 1. Introduction

The incidence of cancer is a matter of great concern on a global scale with increasing incidence and mortality rates placing significant strain on healthcare systems [1]. Cancer is a complex and multifaceted disease driven by genetic and epigenetic dysregulations that result in uncontrolled cell proliferation and immune evasion [2]. Traditional treatment modalities, including surgery, chemotherapy, and radiation therapy, have been the cornerstone of cancer management. Surgery involves the physical removal of cancerous tissue, while chemotherapy and radiation use cytotoxic agents or high-energy rays to kill rapidly dividing cells. While effective in many cases, these approaches often suffer from critical limitations, such as a lack of tumour specificity, toxicity to healthy tissues, and the development of drug resistance, leading to disease recurrence and poor long-term outcomes [3,4]. The urgent need for more targeted, durable, and less toxic treatments has fuelled interest in immunotherapy as a revolutionary approach to cancer care.

Immunotherapy harnesses the body’s immune system to recognise and attack malignant cells, offering the potential for long-term remission in some patients. Immune checkpoint inhibitors, adoptive cell therapies, cancer vaccines, and cytokine-based immunomodulation have shown significant promise in improving survival rates [5,6,7]. However, challenges such as immunotherapy resistance, patient response variability, and immune-related adverse events remain major hurdles [8]. Additionally, the complexity of the tumour microenvironment plays a crucial role in determining treatment success, necessitating novel strategies to enhance immunotherapeutic efficacy.

Recent advancements in gene therapy, epigenetics, and nanotechnology have provided innovative solutions to overcome these challenges. Targeted gene therapies, such as LIM domain only 7 (LMO7) modulation and N6-methyladenosine (m6A) RNA modifications, have been identified as potential regulators of the immune response and tumour progression [9,10]. Nanotechnology-based drug delivery systems have demonstrated an ability to enhance therapeutic precision while minimising systemic toxicity. Multifunctional nanocarriers designed for the co-delivery of chemotherapeutics and gene-silencing agents offer a promising avenue for addressing treatment resistance and tumour heterogeneity [11,12].

Despite these advancements, immunotherapy is not without limitations. Many patients exhibit primary or acquired resistance, and the absence of reliable predictive biomarkers makes patient selection challenging [13,14,15,16]. Moreover, high costs and complex manufacturing processes hinder widespread clinical adoption. Research is increasingly focusing on personalised medicine and combination strategies, integrating immunotherapy with traditional treatments and next-generation technologies to enhance therapeutic outcomes [5,17].

Over the past decade, the FDA has approved a wide range of immunotherapies, including monoclonal antibodies, immune checkpoint inhibitors, and cell-based therapies, many of which have significantly improved patient survival. In this review, we systematically integrate approved immunotherapies with cutting-edge advancements in nanotechnology, personalised medicine, and ongoing clinical trials, providing a comprehensive and sequential perspective on emerging trends and challenges in cancer immunotherapy. By addressing current limitations and exploring innovative strategies, this review aims to contribute to the ongoing effort to refine and expand the potential of immunotherapy in oncology.

## 2. Targeted Antibodies

### 2.1. Monoclonal Antibodies (mAbs)

**Figure 1:**
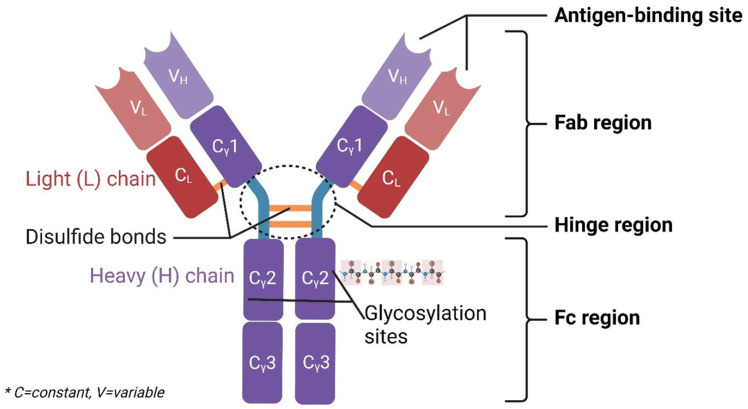
Structural components of immunoglobulin G (IgG). The IgG structure consists of two identical heavy (H) chains and two identical light (L) chains, arranged in a characteristic Y-shape. Key structural elements include the Fab (Fragment antigen-binding) region: Composed of one light chain and part of a heavy chain, containing the antigen-binding sites at the tips of the Y-structure, Fc (Fragment crystallizable) region: Formed by the lower stems of the two heavy chains, responsible for effector functions, Hinge region: A flexible area connecting the Fab and Fc regions, allowing for molecular flexibility, Variable (V) regions: VL in the light chain and VH in the heavy chain, which form the antigen-binding site and confer antigen specificity, and Constant (C) regions: CL in the light chain, and Cγ1, Cγ2, and Cγ3 in the heavy chain, which maintain structural integrity and mediate effector functions.

Monoclonal antibodies are a type of treatment that uses synthetic antibodies to fight cancer. Monoclonal antibody therapy utilises antibodies that result from a single clone of cells, producing identical antibodies with specificity for a particular antigen. This precise targeting makes monoclonal antibody therapy a powerful and potentially more effective treatment option compared with traditional therapies. Among the classes of antibodies used in monoclonal antibody therapy, immunoglobulin G (IgG) is the most employed. As shown in Figure 1, IgG consists of two heavy and light chains, forming a Y-shaped structure [18]. The antigen-binding fragments (Fab) at the ends of the Y are responsible for recognising and binding to specific antigens on target cells. The constant region (Fc) determines effector functions. The interaction between these Fc regions and Fc gamma receptors (FcγRs) plays an integral part in antibody-dependent cellular cytotoxicity (ADCC) and complement-dependent cytotoxicity (CDC) [19]. FcγRs are present in various immune cells, such as leukocytes, dendritic cells, and natural killer (NK) cells. The glycan profile of the Fc region is recognised as a critical quality attribute for therapeutic antibodies, and alterations in Fc glycosylation have been shown to affect the pharmacokinetics and effector functions of mAbs [20,21]. It is also the primary source of post-translational heterogeneity in mAbs, which can significantly impact their mechanism of action (MOA) as a drug [22]. Modifying the Fc portion of IgG1 therapeutic mAbs to regulate the interactions between IgG and receptor FcγRIIIA has emerged as a main focus as FcγRIIIA provides a vital role when assessing the effectiveness of therapeutic mAbs [23,24,25].

**Figure 2:**
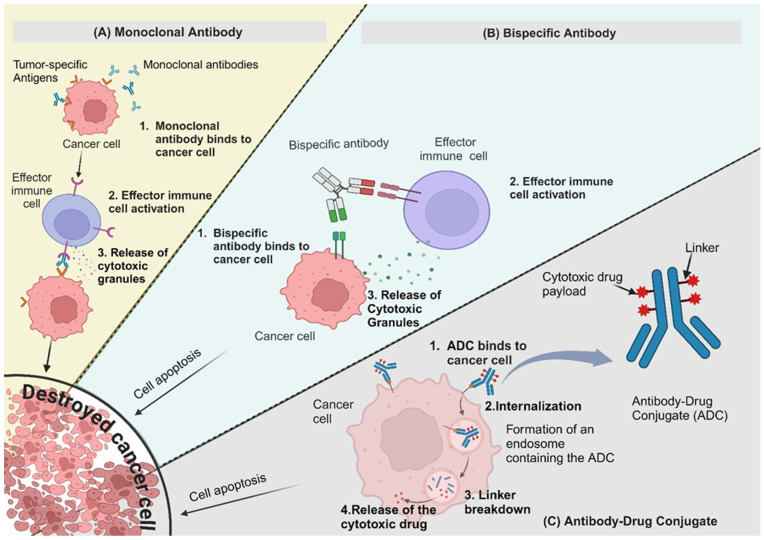
Mechanisms of action of monoclonal antibodies, bispecific antibodies, and antibody–drug conjugates in cancer immunotherapy. (**A**) Monoclonal antibodies: These bind specifically to tumour-associated antigens on cancer cell surfaces, recruiting effector immune cells. The process involves (1) antibody binding to the cancer cell, (2) activation of effector immune cells, and (3) release of cytotoxic granules, leading to cancer cell death. (**B**) Bispecific antibodies: These simultaneously engage tumour antigens and immune cells. The mechanism includes (1) binding to the cancer cell, (2) activation of the effector immune cell through a second binding domain, and (3) release of cytotoxic granules for targeted cell killing. (**C**) Antibody–drug conjugates (ADCs): These combine the specificity of monoclonal antibodies with potent cytotoxic payloads. The process involves (1) ADC binding to cancer cell surface antigens, (2) internalisation of the ADC–antigen complex, (3) breakdown of the linker in the endosome, and (4) intracellular release of the cytotoxic drug, resulting in cancer cell death. These approaches represent key strategies in modern cancer immunotherapy, each leveraging the specificity of antibodies to enhance targeted elimination of malignant cells while potentially reducing off-target effects on healthy tissues.

Figure 2A illustrates one of the primary mechanisms of action, in which mAbs bind directly to cancer cell surface antigens, triggering immune-mediated destruction through processes such as ADCC and CDC. However, not all mAbs function by directly targeting cancer cells. Some, such as vascular endothelial growth factor (VEGF)-targeting antibodies, work by inhibiting tumour angiogenesis, rather than engaging the immune system to attack cancer cells. These antibodies, like Bevacizumab, bind to VEGF, preventing it from interacting with its receptors and thereby disrupting the formation of new blood vessels that support tumour growth. This anti-angiogenic strategy helps to starve the tumour of essential nutrients and oxygen, limiting its progression.

The research and development of mAbs for cancer treatment have advanced significantly, leading to improved patient outcomes and an expanding therapeutic landscape. Clinical trials continue to demonstrate the efficacy of targeted antibodies, highlighting their role in cancer immunotherapy. Alongside these scientific advancements, the market for mAbs has experienced substantial growth, driven by the success of approved therapies and ongoing clinical development. The increasing number of mAbs entering the market reflects their clinical effectiveness and the demand for innovative cancer treatments. Table 1 outlines the success of various mAbs, emphasising their impact on both research progress and commercial expansion. This strong market presence continues to pave the way for further innovation in antibody-based cancer therapies.

### 2.2. Bispecific Antibodies (BsAbs)

Bispecific antibodies (BsAbs) are a significant advancement in antibody therapy, designed to simultaneously bind two targets. This dual-binding capability allows BsAbs to connect distinct cell types or pathways in tumour progression, enhancing therapeutic efficacy. Figure 2B illustrates the mechanisms by which BsAbs function, demonstrating how they facilitate immune cell engagement and tumour cell targeting.

One clinically approved BsAbs is BLINCYTO (blinatumomab), a bispecific T-cell engager (BiTE) that dually binds CD19 on B cells and CD3 on T cells. BLINCYTO received FDA approval in 2014 for relapsed/refractory B-cell acute lymphoblastic leukaemia (B-ALL). In June 2024, it gained additional approval for consolidation treatment of newly diagnosed CD19-positive Philadelphia chromosome-negative B-ALL in adult and paediatric patients. The phase III E1910 trial showed that adding BLINCYTO to multiphase consolidation chemotherapy reduced the risk of death by 58% compared with chemotherapy alone, with five-year overall survival rates of 82.4% versus 62.5%. This makes BLINCYTO the first and only BiTE therapy approved for frontline B-ALL consolidation, regardless of measurable residual disease status.

Several BsAbs are in phase III clinical trials, including SI-B001, AK112, KN046, SHR-1701, CTX-009, PM8002, M7824, and Elranatamab. These drugs have shown promise in pre-clinical and early clinical trials. SI-B001 is a bispecific antibody targeting EGFR and HER3, two receptors commonly overexpressed in certain cancers [26]. By simultaneously inhibiting these pathways, SI-B001 aims to enhance anti-tumour activity and overcome resistance mechanisms. A Phase II/III clinical trial (NCT05020769) is evaluating its efficacy in combination with osimertinib mesylate for treating recurrent and metastatic non-small-cell lung cancer (NSCLC). SHR-1701 is another promising bispecific therapeutic, designed as a bifunctional fusion protein targeting PD-L1 and TGF-β. This dual-targeting approach modulates the tumour microenvironment by both enhancing immune activation and reducing immunosuppressive signalling. SHR-1701 is currently being investigated in a Phase Ib clinical trial (NCT04282070) for patients with recurrent or metastatic nasopharyngeal carcinoma [27]. Similarly, HLX301 is a recombinant humanised anti-PDL1 and anti-TIGIT bispecific antibody. The Phase I/II clinical trial (NCT05102214) aims to evaluate its safety, tolerability, pharmacokinetics, and anti-tumour efficacy in patients with locally advanced or metastatic solid malignancies [28]. Another well-advanced BsAb is Elranatamab, which targets BCMA on multiple myeloma cells and CD3 on T cells, redirecting T cells to attack cancer cells. The Phase II MagnetisMM-3 trial (NCT04649359) is currently assessing its effectiveness in relapsed or refractory multiple myeloma. This bispecific approach has shown significant potential in engaging the immune system for targeted elimination of cancer cells [29]. Additionally, several other BsAbs, including AK129, AK117, AK112, HLX301, EMB-01, EMB-02, EMB-09, PM8002, AZD2936, IMM2902, MCLA-128, MCLA-129, and KN026, are currently undergoing early clinical trials, demonstrating promise in the treatment of advanced malignant tumours, with many trials focusing NSCLC. Regardless of the considerable advances in BsAb, researchers are also focused on multispecific antibodies engineered to simultaneously target multiple antigens and enhance their ability to recognise and engage cancer cells. These antibodies can target both tumour cells and immune cells, leading to improved specificity and efficacy [30]. Ongoing research in the field of multispecific antibodies is focused on optimising their design, stability, and pharmacokinetics to enhance their therapeutic potential. For instance, researchers are exploring different formats of multispecific antibodies, such as bispecific T-cell engagers (BiTEs) and tri-specific antibodies, to enhance their ability to engage immune cells and improve tumour targeting [30,31,32,33,34].

### 2.3. Antibody-Drug Conjugates (ADCs)

Another noteworthy aspect of research is the development of antibody–drug conjugates. ADCs merge the selectivity of antibodies with the potency of cytotoxic drugs, resulting in highly targeted and effective therapies. Figure 2C illustrates the mechanism by which ADCs deliver cytotoxic payloads to tumour cells, ensuring precise targeting while minimising off-target effects.

Scientists are engineering next-generation antibodies with even greater affinity and selectivity for cancer-specific targets, enabling ADCs to pinpoint specific tumour cells with laser-like precision, minimising off-target effects and side effects. Among the many exciting ADCs currently under investigation are those exploring diverse targets and modalities. Some ADCs recognise unique markers on cancer cells. For example, sacituzumab govitecan (Trodelvy) targets trophoblast cell surface antigen 2 (Trop-2) in triple-negative breast cancer, belantamab mafodotin (Blenrep) targets B-cell maturation antigen (BCMA) in multiple myeloma, and trastuzumab deruxtecan (T-DXd) targets human epidermal growth factor receptor 2 (HER2) in breast cancer [35,36,37,38] while researchers are developing engineered ADCs to efficiently penetrate tumour tissues and deliver cytotoxic payloads to cancer cells. These ADCs are engineered to enhance tissue penetration, intracellular drug release, and overall therapeutic efficacy. It holds promise for treating tumours that lack specific surface markers for targeted ADCs [39,40]. Scientists are currently investigating the potential of combining antibodies and ADCs with radiation therapy to enhance the effectiveness of targeted therapy. Recent studies conducted by Lewis et al. [41] and Singh, Dadey, Rau, Fitzpatrick, Shah, Saikia, Townsend, Thotala, Hallahan and Kapoor [31] have demonstrated the promising results of utilising radiation-inducible antigens to enhance the efficacy of antibodies and antibody–drug conjugates.

### 2.4. Antibody–Oligonucleotide Conjugates (AOCs)

AOCs are a type of molecule that possess dual functionality. They can target specific molecules while also possessing the hybridisation and recognition capabilities of oligonucleotides. [42]. Researchers are investigating the use of AOCs for targeted drug delivery, gene regulation, and imaging in cancer therapy [43,44]. The conjugation of antibodies with oligonucleotides allows the targeted delivery of therapeutic payloads to cancer cells, thereby reducing the off-target effects. Another exciting development is the use of intracellular antibody fragments also known as single-chain variable fragments (scFvs), which are engineered fragments of antibodies that are designed to penetrate the cell membrane and target intracellular antigens. Researchers are exploring the use of scFvs to block intracellular targets involved in cancer cell survival, proliferation, and metastasis and delivery methods, like cell-penetrating peptides, to ensure effective and targeted delivery of scFvs [45,46,47,48]. This approach holds promise for targeting antigens not accessible on the cell surface. These innovative approaches highlight a multifaceted strategy to address the complexity of tumours, emphasising the continuous pursuit of advancements in design, delivery, and combination therapies for improved patient outcomes.

## 3. Immune Checkpoint Inhibitors (ICIs)

Immune checkpoint inhibitors have gained significant attention in the treatment of various cancers over the past few decades. These drugs work by blocking immune checkpoints that regulate immune responses. Immune checkpoints prevent the immune system from attacking healthy cells in the body. However, cancer cells can manipulate these checkpoints to avoid detection by the immune system. By blocking these checkpoints, checkpoint inhibitors enable the immune system to recognise and attack cancer cells [49].

One of the most well-characterised immune checkpoints is cytotoxic T-lymphocyte-associated protein 4 (CTLA-4). CTLA-4 is expressed on activated T cells, competing with CD28 for binding to B7 molecules (CD80/CD86) on antigen-presenting cells (APCs). Unlike CD28, which provides a stimulatory signal for T cell activation, CTLA-4 transmits an inhibitory signal, suppressing T cell proliferation and dampening the immune response. CTLA-4 inhibitors block this inhibitory interaction, leading to enhanced T-cell activation and tumour destruction [50,51]. Another critical checkpoint pathway involves programmed cell death protein 1 (PD-1) and its ligands PD-L1 and PD-L2. PD-1 is expressed on activated T cells, B cells, and natural killer (NK) cells, while PD-L1 is often overexpressed on tumour cells and immune-suppressive cells within the tumour microenvironment. When PD-1 binds to PD-L1, it inhibits T-cell function, allowing cancer cells to evade immune detection. PD-1 inhibitors, such as Nivolumab (Opdivo) and Pembrolizumab (Keytruda), and PD-L1 inhibitors, such as Atezolizumab (Tecentriq) and Durvalumab (Imfinzi), disrupt this interaction, restoring T cell function and promoting anti-tumour immunity [52]. Beyond CTLA-4 and PD-1/PD-L1, researchers are actively investigating additional immune checkpoint targets to overcome resistance to existing ICIs. Lymphocyte activation gene-3 (LAG-3) is an inhibitory receptor expressed on exhausted T cells and regulatory T cells (Tregs) [53]. LAG-3 negatively regulates T cell proliferation and immune responses. TIGIT (T cell immunoreceptor with Ig and ITIM domains) is another inhibitory molecule that binds CD155 on tumour cells and dendritic cells, suppressing T cell activation [54]. VISTA (V-domain Ig suppressor of T cell activation), expressed on myeloid-derived suppressor cells (MDSCs) and T cells, functions as a negative regulator of T cell responses [55]. Inhibitors targeting these checkpoints are currently under clinical investigation and show potential in enhancing immune responses against tumours. Another promising immune checkpoint target is indoleamine 2,3-dioxygenase (IDO), an enzyme that degrades tryptophan, producing immunosuppressive metabolites that hinder T cell proliferation and activity. Tumours frequently upregulate IDO to create an immune-suppressive microenvironment, allowing them to escape immune surveillance. Inhibitors of IDO, in combination with PD-1/PD-L1 blockade, are currently being evaluated in clinical trials to determine their potential to boost anti-tumour immunity [56,57].

The clinical success of ICIs has revolutionised cancer immunotherapy, leading to the FDA approval of multiple checkpoint inhibitors for various malignancies, as summarised in Table 2, including melanoma, non-small-cell lung cancer (NSCLC), urothelial carcinoma, and Hodgkin’s lymphoma. However, challenges remain, including immune-related adverse events (irAEs), such as colitis, endocrinopathies, and pneumonitis, which necessitate careful patient monitoring and management [58,59,60].

Ongoing research aims to optimise combination strategies, such as ICIs with chemotherapy, radiotherapy, and targeted therapies, to enhance efficacy while mitigating toxicity [61,62,63,64,65]. In fact, over 70 clinical trials have been registered since 2020 on ClinicalTrials.gov, exploring the effectiveness and safety of these combinations in various cancer types. Furthermore, researchers are also exploring the application of immunotherapy in conjunction with other targeted therapeutics, like poly (ADP-ribose) polymerase (PARP) inhibitors and anti-angiogenics [66]. For example, the combination of Lenvatinib and Pembrolizumab has shown remarkable results in patients with advanced solid tumours, including endometrial cancer, renal cell carcinoma, and NSCLC in phase I and II trials [67], and a recent trial, KEYLYNK-009, is studying the effectiveness of Olaparib, a PARP inhibitor, plus Pembrolizumab in treating triple-negative breast cancer (TNBC) [65]. Moreover, small molecules have been investigated for their potential to modulate kinase signalling pathways and combine with immune checkpoint inhibitors, leading to enhanced antitumour effects and improved survival in preclinical studies [68,69,70]. The potential of immune biomarkers in meningiomas and thymic epithelial tumours has been explored and a high prevalence of PD-L1 expression has been shown in anaplastic meningioma, B3 Thymoma (TB3), and Thymic Carcinoma (TC), and researchers have identified CD4 and CD8 single-positive lymphocytes as potential targets for immunotherapy [71,72,73,74]. ICIs represent a significant advancement in cancer treatment, particularly in the context of activating the immune response against cancer cells. Their efficacy is demonstrated in several types of cancer, and their combination with other therapeutic modalities shows promise in improving treatment outcomes. However, the administration of ICIs has resulted in the occurrence of immune-related adverse events (irAEs), such as endocrinopathies and checkpoint inhibitor pneumonitis (CIP), in the skin, endocrine, digestive tract, and lungs [75,76]. By expanding our understanding of immune checkpoint pathways and developing next-generation inhibitors, immunotherapy continues to evolve, offering personalised and effective treatments for a growing number of cancer patients.

### Nanocarrier-Based Delivery of Immune Checkpoint Inhibitors

The use of nanocarriers for the delivery of ICIs offers a promising approach to enhancing the efficacy of cancer immunotherapy. By improving the pharmacokinetics, biodistribution, and local concentration of these inhibitors, nanocarriers can optimise their therapeutic potential while minimising systemic toxicity. One significant study by Chen et al. [77] explored biodegradable PEG-poly(ω-pentadecalactone-co-p-dioxanone) nanoparticles for sustained drug delivery in brain tumours. Their research demonstrated that such nanocarriers could enhance the retention and distribution of therapeutic agents, which are crucial for checkpoint inhibitors that require prolonged exposure to exert their effects. The sustained release profile of these nanoparticles allows for a more effective blockade of the PD-1/PD-L1 interaction, which is essential for reversing T-cell exhaustion in the tumour microenvironment [78]. Moreover, the combination of checkpoint inhibitors with other therapeutic modalities has been investigated using nanocarrier systems. For instance, a study by Chen et al. [79] highlighted the potential of co-delivering immunomodulatory agents alongside chemotherapeutics using a PEG-based nanocarrier. This approach not only enhances the immune response but also addresses the challenge of myeloid-derived suppressor cell accumulation, which can limit the efficacy of immunotherapy. The ability of nanocarriers to encapsulate multiple agents allows for a synergistic effect, enhancing the overall therapeutic outcome. The clinical implications of PD-1/PD-L1 inhibitors are underscored by their ability to improve patient outcomes in various cancers, as seen in trials involving nivolumab and pembrolizumab. These agents have shown significant efficacy in advanced non-small-cell lung cancer and urothelial carcinoma, respectively [80,81]. However, the variability in PD-L1 expression among tumours complicates the predictive value of these biomarkers for treatment response [82]. Nanocarrier systems can potentially address this issue by providing targeted delivery to tumours with low PD-L1 expression, thereby broadening the applicability of checkpoint inhibitors. Additionally, the use of nanocarriers can mitigate the adverse effects associated with the systemic administration of checkpoint inhibitors. For example, immune-related adverse events (IRAEs) are a significant concern with checkpoint blockade therapies [83]. By utilising targeted nanocarriers, it may be possible to reduce off-target effects and enhance the localised action of these inhibitors within the tumour microenvironment, thereby minimising systemic toxicity and improving patient tolerability [84]. The integration of nanocarrier technology in the delivery of checkpoint inhibitors holds great promise for enhancing the efficacy of cancer immunotherapy. By improving drug delivery, enabling combination therapies, and reducing systemic side effects, nanocarriers can significantly contribute to the advancement of immunotherapeutic strategies in oncology.

## 4. Cancer Vaccines

Cancer vaccines are a type of immunotherapy that aims to train the immune system to recognise and attack cancer cells. There are two main types of cancer vaccines: preventive (prophylactic) vaccines and therapeutic vaccines [85]. Preventive vaccines aim to prevent certain types of cancer by targeting viruses known to cause cancer, such as the human papillomavirus (HPV) vaccine for cervical cancer [86]. Therapeutic vaccines, on the other hand, are designed to treat existing cancer by stimulating the immune system to recognise and attack cancer cells.

**Figure 3:**
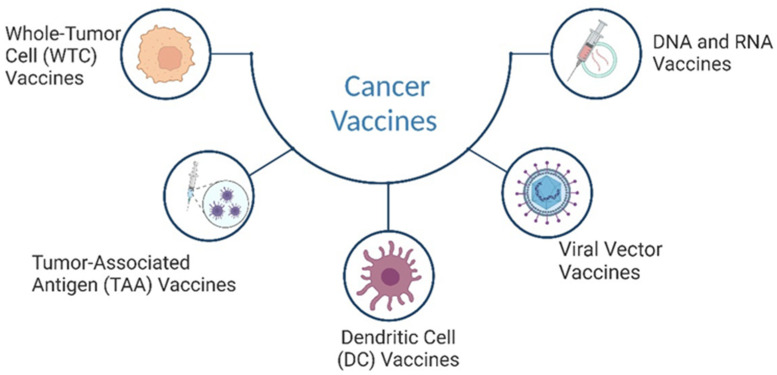
Classification of cancer vaccines based on the source and mechanism of action. (1) Associated Antigen (TAA) Vaccines: target specific proteins expressed by cancer cells; (2) Whole-Tumour Cell (WTC) Vaccines: derived from entire cancer cells that have been inactivated; (3) Dendritic Cell (DC) Vaccines: utilising patient-derived dendritic cells loaded with cancer antigens; (4) Viral Vector Vaccines: employing modified viruses to deliver cancer antigens; and (5) DNA and RNA Vaccines: using genetic material encoding cancer antigens. Each type represents a distinct approach to stimulating the immune system against cancer cells, offering various strategies for both preventive and therapeutic applications in cancer immunotherapy.

These vaccines can be categorised into several types based on their composition and mechanism of action. This review focuses on five main categories of cancer vaccines: tumour-associated antigen (TAA) vaccines, whole-tumour cell (WTC) vaccines, dendritic cell vaccines, viral vector vaccines, and DNA/RNA vaccines (Figure 3). Each type employs a unique strategy to stimulate an immune response against cancer cells, offering diverse approaches to cancer immunotherapy. Tumour-associated antigen (TAA) vaccines: TAA is a type of cancer vaccine designed to stimulate the immune system to recognise and target antigens expressed by cancer cells. These vaccines aim to induce an immune response against specific proteins or peptides found on the surface of cancer cells, known as tumour-associated antigens (TAAs) [87,88]. The immune response elicited by TAA vaccines can lead to the generation of cytotoxic T lymphocytes (CTLs) and the production of antibodies that target and eliminate cancer cells [89]. Whole-tumour cell vaccines (WTC): these vaccines are made from entire cancer cells that have been killed or weakened.

When injected into the body, these vaccines trigger an immune response against cancer cells, stimulating the production of antibodies and T cells that can recognise and attack cancer cells throughout the body [90]. WTC has been modified to overexpress stimulatory molecules, such as fibroblast-activation protein (FAP), granulocyte-macrophage colony-stimulating factor (GM-CSF), and CD86, to confer significant antitumour effects [91]. Additionally, WTC has been used as the source of tumour antigens for the development of dendritic cell vaccines [92]. Dendritic cell vaccines; dendritic cells are specialised immune cells that play a crucial role in activating other immune cells. Dendritic cell vaccines are created by taking a dendritic cell from patients, loading them with cancer antigens, and then injecting them back into the patient. This helps to prime the immune system to attack cancer cells [93,94]. Viral vector vaccines: these vaccines use viruses that have been modified to carry cancer antigens. When injected into the body, the viruses infect cells and deliver the antigens, stimulating an immune response against the cancer cells [95]. DNA and RNA vaccines; these vaccines use DNA or RNA molecules that encode cancer antigens. When injected into the body, the genetic material enters cells and instructs them to produce antigens, stimulating an immune response against the cancer cells [96]. Cancer vaccines are an active area of research and scientists are constantly working to improve their effectiveness and overcome challenges. With continued progress, cancer vaccines have the potential to revolutionise cancer treatment and provide new hope for cancer patients. However, not many cancer vaccines have been approved for cancers. According to Grimmett et al. [97], Table 3 shows the existing cancer vaccines that are approved for clinical use. Although cancer vaccines have potential benefits, their efficacy has been limited due to challenges in identifying appropriate antigens and the tumour microenvironment creating barriers to the immune response [98]. However, ongoing research is focused on combining cancer vaccines with other immunotherapies, such as immune checkpoint inhibitors, to improve their efficacy. Despite limitations, cancer vaccines remain a promising tool in the fight against cancer, and further research aims to refine their role in treatment [99,100].

There are numerous clinical trials underway evaluating the efficacy and safety of various cancer vaccines for the treatment of different cancers. These trials are exploring the use of cancer vaccines in combination with other therapies, such as chemotherapy or radiation, to enhance treatment outcomes. One area of focus is the development of personalised cancer vaccines. These vaccines are designed to target specific mutations or antigens found in the patient’s cancer cells. This allows for a more targeted and effective treatment. Ongoing trials are investigating the efficacy of personalised cancer vaccines in various cancers, including breast cancer, lung cancer, and melanoma. However, a recent phase II clinical trial with personalised immunotherapy based on tumour lysate-charged dendritic cell vaccination failed to prolong survival in glioblastoma patients [101]. Despite this setback, other personalised immunotherapy approaches have shown success. One such approach is the ChAdOx1-MVA 5T4 vaccine, which has an excellent safety profile and has been shown to induce T-cell responses in early stage prostate cancer patients [102]. Another promising approach is a combination treatment of radiofrequency excision and neoantigen vaccination, which has been shown to improve clinical and immune response in cancer patients [103]. In an early phase II study, a mixed 19-peptide cancer vaccine monotherapy showed safety and immune boosting in advanced triple-negative breast cancer patients in refractory to systemic chemotherapy. This study highlights the potential of personalised immunotherapy in treating difficult-to-treat cancers [104]. Similarly, the P10s-PADRE vaccine combined with standard-of-care chemotherapy in HR+/HER2 early stage breast cancer patients has been shown to be safe and immunogenic [105]. Additionally, iNeo-Vac-P01 as monotherapy has been found to be feasible and safe for patients with advanced solid tumours, such as pancreatic cancer, showing promising antitumour efficacy [106,107]. DCP-001, a novel dendritic cell vaccine derived from leukaemia cells, has shown promise in treating acute myeloid leukaemia (AML). In a phase II study, the vaccine successfully converted Measurable residual disease (MRD) positive AML patients in the first complete remission to negative, resulting in deeper remissions. MRD refers to the presence of residual cancer cells that cannot be detected by conventional morphological evaluation [108]. Moreover, a clinical study conducted by Sahin et al. [109] shows the melanoma FixVac (BNT111) RNA vaccine, an intravenously administered liposomal RNA (RNA-LPX) vaccine, targeted towards four melanoma-associated antigens produces durable objective responses. It has demonstrated lasting objective responses in clinical trials for melanoma patients. These responses are supported by robust CD4+ and CD8+ T-cell immunity, with CD4+ T-cells coordinating the immune response and CD8+ T-cells attacking and eliminating cancer cells. The recent clinical trials underscore the ongoing progress in cancer vaccine research. The development of personalised cancer vaccines and vaccines that are combined with other therapies is leading to more effective and targeted treatments for various cancers, offering hope for patients with advanced or metastatic disease.

### Nanocarrier-Based Approaches for Cancer Vaccine Delivery

The integration of nanocarriers in the development of cancer vaccines has become a focal point of recent research, owing to their potential to significantly enhance the targeting, efficacy, and specificity of cancer immunotherapies. Various nanocarrier platforms have been explored for their ability to effectively deliver antigens and adjuvants to the immune system, thus amplifying the immune response against tumours.

One notable strategy involves the use of peptide nanoclusters, as exemplified by Tsoras and Champion [110], who developed cross-linked peptide nanoclusters (PNC) for the delivery of oncofoetal antigens. Their research demonstrates that these nanocarriers can effectively activate immune cells, potentially offering a novel approach to therapeutic cancer vaccines. Similarly, Jiang et al. [111] investigated micelle-tailored vaccines that facilitate the cytosolic delivery of antigens, which is crucial for activating CD8+ T cells and generating a robust immune response against tumours. These studies underscore the importance of antigen delivery to the cytoplasm to elicit strong immune activation.

In addition to peptide-based systems, other nanocarrier platforms, such as lipid-based nanoparticles and polymeric nanocarriers, have shown great promise for cancer vaccine delivery. The review by Sunoqrot et al. [112] highlights the advancements in lipid- and polymer-based nanocarriers, emphasising their role in enhancing vaccine immunogenicity and overall efficacy. Zhang et al. [113] further contributed to this field by exploring the design of nanovaccines that utilise both synthetic and biogenic nanocarriers, which improve targeting and therapeutic effectiveness in cancer immunotherapy.

A particularly innovative approach involves the use of outer-membrane vesicles (OMVs) derived from bacteria, as demonstrated by Li et al. [114], who showed that OMVs are highly effective for displaying and delivering mRNA antigens for personalised tumour vaccines. The natural properties of OMVs enhance immune responses to specific tumour antigens, providing a promising strategy for cancer vaccine development. Furthermore, small extracellular vesicles (sEVs) have gained attention as promising nanocarriers for cancer vaccines, due to their ability to aid in early diagnosis and treatment monitoring, while also serving as efficient delivery systems for antigens [115].

Targeting strategies are also integral to improving the efficacy of cancer vaccines. Simon et al. [116] emphasised the importance of site-directed conjugation of targeting antibodies to nanocarriers, enabling specific delivery to dendritic cell subsets. This targeted approach helps to improve the induction of antigen-specific immune responses. In a similar vein, Wang et al. [117] noted that nanocarriers can activate both innate and tumour-specific immunity, thereby enhancing the overall therapeutic effect of cancer vaccines.

Another promising avenue is the use of stimuli-responsive polymeric nanovaccines, which enable the controlled delivery of antigens and adjuvants to immune cells while preventing premature degradation. This capability is crucial for sustaining adaptive immune responses and enhancing the effectiveness of immunotherapy [118]. The versatility of nanocarriers is further exemplified by the development of hybrid systems that combine lipid and polymer-based platforms, which have been shown to improve both the delivery and efficacy of cancer vaccines [112]. The exploration of various nanocarrier platforms for cancer vaccine delivery has led to significant advancements in cancer immunotherapy. Ongoing research continues to uncover innovative strategies that improve the specificity, efficacy, and safety of cancer vaccines, opening the door to more effective treatment modalities for oncology.

## 5. Cytokines

Cytokine treatments for cancer have been an area of active research and development. Ongoing research continues to explore the potential of cytokines in combination with other therapies and in specific patient populations. The development of novel cytokine variants and delivery systems is also being investigated to improve their efficacy and reduce side effects [119,120]. Currently, the FDA has only approved a few cytokine treatments for cancer, which include: interferon alpha (IFN-α): IFN-α was approved for treating hairy-cell leukaemia (HCL) in 1986. This was the first cytokine to be approved for cancer treatment [121]. Since then, it has been used in the treatment of various malignancies, including hepatocellular carcinoma (HCC), malignant melanoma, and renal cell cancer (RCC). It has been shown to promote apoptosis and inhibit the growth of several tumour cell types, which makes IFNα a promising treatment option for cancer patients [122]. High-dose interleukin-2 (HDIL-2): HDIL-2 is approved for the treatment of metastatic renal cell carcinoma (mRCC), and metastatic melanoma (MM). It was first approved for mRCC in 1992 and for MM in 1998 [121]. IL-2 enhances the natural ability of the immune system to kill tumours by increasing the tumouricidal activity of the cytotoxicity of CD8+ T cells and natural killer (NK) cells [123]. Cytokine-induced killer (CIK) cell immunotherapy has emerged as a promising treatment approach for various types of cancer. In recent years, several studies have shown the potential benefits of combining CIK cell immunotherapy with chemotherapy in patients with advanced squamous non-small-cell lung cancer (NSCLC). CIK cells can enhance the antitumour activity of chemotherapy by targeting cancer cells resistant to chemotherapy. Liu et al. [124] investigated the efficacy and safety of CIK cell immunotherapy combined with chemotherapy in patients with previously untreated, advanced squamous NSCLC. The phase II results of the study showed that this combination therapy improved the efficacy of chemotherapy and had a good safety profile. Based on these findings, a large sample, multi-centre randomised, phase III trial is currently being carried out to further validate these results. Inflammatory cytokines have also been shown to play important roles in cancer treatment. For instance, Crake et al. [125] reported that monocyte chemoattractant protein 1 inversely correlates with CYP3A4 (Cytochrome P450 enzyme) activity during breast cancer chemotherapy. This suggests that inflammatory cytokines may affect the metabolism and efficacy of chemotherapy drugs. Similarly, Safi et al. [126] found that preoperative serum interleukin-4 levels can predict T-cell responses specific for tumour-associated antigens and recurrence-free survival in non-small-cell lung cancer patients. These findings highlight the potential of using inflammatory cytokines as biomarkers for predicting treatment outcomes in cancer patients. These studies hold promise for improving cancer treatment and patient outcomes. As research continues, we can expect further advancements in these areas, leading to more effective and personalised treatment strategies.

## 6. Oncolytic Viruses

Oncolytic viruses represent a novel and promising frontier in cancer treatment. These are viruses that have been engineered or naturally occur in a way that allows them to selectively infect and kill cancer cells while leaving healthy cells largely unharmed [127]. The oncolytic virus approach provides a unique dual mechanism of action—not only do these viruses directly lyse tumours from within, but they can also stimulate the body’s immune defences against cancer [128].

While only one oncolytic virus, Talimogene Laherparepvec (T-VEC), an oncolytic herpes simplex virus type 1 (HSV-1), has received FDA approval so far for treating inoperable melanoma, an array of other candidates have been evaluated in clinical trials across various solid tumours and haematological malignancies. T-VEC achieves tumour specificity by deleting viral genes encoding ICP34.5 and ICP47, which are responsible for neurovirulence and immune evasion in normal tissues. Cancer cells, which often have defective antiviral responses, allow T-VEC to replicate and induce lysis, while the deletion of ICP47 enhances immune recognition by promoting antigen presentation [129]. In a Phase III trial, T-VEC demonstrated improved durable response rates compared with granulocyte–macrophage colony-stimulating factor (GM-CSF), leading to its FDA approval for treating inoperable melanoma [130,131]. Another oncolytic herpes virus, HF10, is naturally occurring and lacks certain neurovirulence-associated genes, making it highly selective for tumour cells. Unlike T-VEC, HF10 retains its replication ability in normal cells, but shows preferential infectivity in cancer cells due to their defective antiviral defences. Early phase clinical trials of HF10 demonstrated a favourable safety profile with no dose-limiting toxicities. A Phase II trial investigated its use in combination with ipilimumab for advanced melanoma, while a Phase I study explored HF10 alongside chemotherapy for pancreatic cancer, aiming to determine optimal dosing regimens. Moreover, Teserpaturev/G47Δ (Delytact), another oncolytic HSV-1, is a triple-mutated virus with deletions in ICP34.5, ICP6, and α47 genes, reducing neurovirulence and enhancing tumour selectivity. It has received approval in Japan for the treatment of malignant glioma and is being evaluated in trials for prostate cancer, mesothelioma, and neuroblastoma [132]. The adenovirus-based candidate Oncorine (H101) is an oncolytic adenovirus developed by Shanghai Sunway Biotech Co., Ltd. (Shanghai, China), that was approved in 2005 by Chinese regulators for the treatment of nasopharyngeal carcinoma in combination with chemotherapy, becoming the world’s first approved oncolytic virus therapy for cancer. H101 is an engineered adenovirus with deletions in the E1B-55kDa gene, allowing it to replicate selectively in p53-deficient tumour cells while sparing normal cells [133]. After successful Phase III trials, it achieved GMP certification in 2006, paving the way for the commercial production of this pioneering oncolytic virus product [134,135]. Two clinical trials are investigating oncolytic adenoviruses in combination therapies: Oncorine (H101) with a PD-1 inhibitor and chemotherapy for advanced non-small-cell lung cancer, and ONCOS-102 with low-dose cyclophosphamide for refractory solid tumours. Both trials underscore the potential of oncolytic adenoviruses to enhance anti-tumour effects while ensuring safety in cancer treatment. Furthermore, Pexa-Vec (JX-594) is a promising oncolytic vaccinia virus engineered to express GM-CSF and β-galactosidase, which enhance immune stimulation and facilitate tumour cell targeting. It selectively replicates in tumour cells due to their reliance on activated Ras signalling, making it effective against many solid tumours [136]. It is being extensively evaluated across various solid tumour types through multiple clinical trials exploring different administration routes, dosing regimens, and combination strategies. A key focus has been leveraging Pexa-Vec’s unique ability to induce immunogenic tumour cell death and stimulate anti-tumour immunity when combined with immune checkpoint inhibitors. One of the pivotal trials is a Phase I/II study investigating Pexa-Vec in combination with the checkpoint inhibitors tremelimumab and Durvalumab for metastatic colorectal cancer refractory to standard therapies. By combining Pexa-Vec’s oncolytic effects with the immune-modulating actions of these antibodies, researchers aim to overcome the immunosuppressive tumour microenvironment and elicit potent anti-tumour responses. Additionally, Pexa-Vec is being explored in the paediatric setting through a Phase I dose-escalation trial for various refractory solid tumours in children and adolescents. Beyond combination regimens, several trials are evaluating Pexa-Vec as a monotherapy, delivered either intratumorally or intravenously, across different solid tumour types, like colorectal cancer, hepatocellular carcinoma, melanoma, and others. These studies aim to characterise Pexa-Vec’s safety profile, optimal dosing, and potential anti-tumour activity, laying the groundwork for future trials and combination approaches. With its unique mechanisms of action and potential for synergy with other treatment modalities, Pexa-Vec represents a promising oncolytic virus candidate that could potentially revolutionise cancer therapy. Another promising oncolytic vaccinia virus candidate is GL-ONC1, also known as Olvi-Vec, which is being actively evaluated for ovarian cancer. A pivotal Phase III trial is currently underway, investigating the efficacy and safety of using GL-ONC1 followed by platinum-based doublet chemotherapy and bevacizumab, compared with chemotherapy and bevacizumab alone in patients with platinum-resistant or refractory ovarian, fallopian tube, or primary peritoneal cancers. This randomised study holds promise for the potential incorporation of an oncolytic virus into standard treatment regimens for these difficult-to-treat gynaecological cancers. The oncolytic reovirus pelareorep is also being investigated for triple-negative breast cancer (TNBC). Pelareorep exploits tumour cells’ defective antiviral response, particularly their impaired PKR (protein kinase R) pathway, which normally suppresses viral replication in healthy cells [137]. A Phase II study is evaluating pelareorep in combination with the PD-1 inhibitor retifanlimab for patients receiving second- or third-line treatment for metastatic TNBC. The key objectives are to assess the anti-tumour activity and safety profile of pelareorep in combination with retifanlimab. Clinical trials have demonstrated the potential of oncolytic viruses as a new modality for cancer treatment, either as monotherapies or in rational combinations with other agents. While challenges remain, the field continues to make steady progress, offering hope for more effective and targeted therapies for cancer patients.

## 7. Adoptive Cell Therapy

### 7.1. Chimeric Antigen Receptor T (CAR-T) Cell Therapy

**Figure 4:**
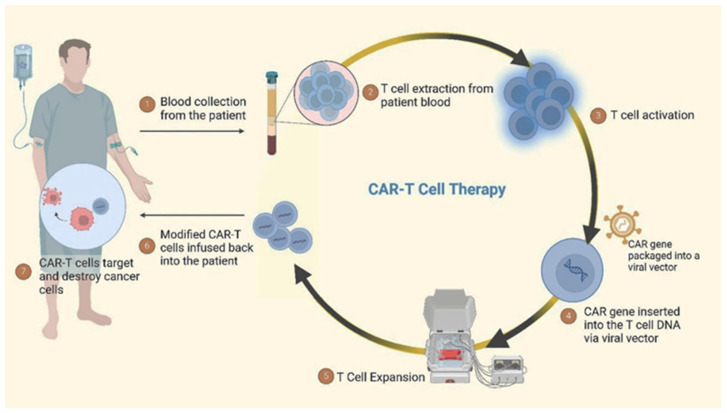
Schematic representation of the Chimeric Antigen Receptor (CAR) T cell therapy for cancer treatment. CAR-T cell therapy involves genetically modifying a patient’s T cells to express CARs that enable them to specifically target and destroy cancer cells. The process begins with (1) blood collection from the patient. (2) T cells are isolated from the collected blood. (3) The extracted T cells are activated and prepared for genetic modification. (4) The CAR gene is usually inserted into the T cell DNA using a viral vector. This modified gene enables T cells to recognise and target cancer cells. (5) The genetically modified T cells (CAR-T cells) are then expanded in culture to produce sufficient numbers for treatment. (6) The expanded CAR-T cells are infused back into the patient, where (7) the infused CAR-T cells circulate in the patient’s body, recognising and destroying cancer cells expressing the target antigen.

**Figure 5:**
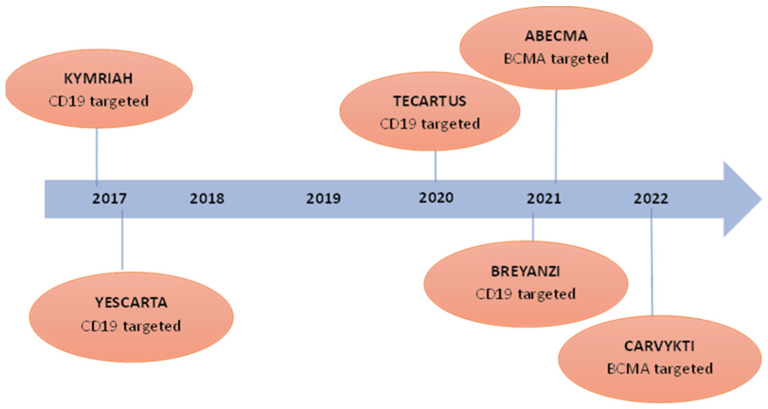
Timeline of FDA-approved CAR-T cell therapy drugs for blood cancer and their corresponding drug targets.

CAR-T cell therapies have shown promising results in treating cancers and have transformed the approach to addressing cancer. The evolution of CAR-T cell therapies has been a result of significant advancements in genetic engineering and immunology [138,139]. A schematic representation of CAR-T cell therapy for cancer treatment is shown in Figure 4. CAR-T cell therapy includes obtaining T cells from the patient and their subsequent modification in a laboratory to express CARs capable of identifying and attacking cancer cells. The engineered T cells are then reintroduced into the patient’s body via infusion, making them capable of identifying and destroying cancer cells. The market for CAR-T cell therapies has witnessed substantial growth, and several CAR-T cell therapies have received FDA approval for blood cancers, as shown in Figure 5. KYMRIAH (tisagenlecleucel) is approved for the treatment of adult patients with relapsed or refractory follicular lymphoma after two or more lines of systemic therapy. It targets CD19, a protein that is expressed on the surface of B cells and tumours derived from B cells, and this therapy was manufactured by Novartis Pharmaceuticals Corporation and approved by the FDA in August 2017. FDA granted regular approval to YESCARTA (axicabtagene ciloleucel) in October 2017. The therapy was manufactured by Kite Pharma, USA and approved for the treatment of adults with relapsed or refractory diffuse large B-cell lymphoma (DLBCL), and approval has been granted for patients experiencing refractory and recurrent conditions or whose cancer remains unresponsive to alternative treatments. It also targets CD19. TECARTUS (brexucabtagene autoleucel) is approved for the treatment of adults with relapsed or refractory mantle cell lymphoma (r/r MCL) or B-cell acute lymphoblastic leukaemia (B-ALL). This therapy is a second-generation CAR-T cell therapy that also targets CD19. The drug was manufactured by Kite Pharma and approved by the FDA in July 2020. BREYANZI (lisocabtagene maraleucel) is another CD19 targeted therapy and approved for the treatment of adults with large B-cell lymphoma (LBCL), including diffuse large B-cell lymphoma (DLBCL), high-grade B-cell lymphoma, primary mediastinal large B-cell lymphoma, and follicular lymphoma grade 3B, and is manufactured by Juno Therapeutics, Inc. The therapy was approved by the FDA in February 2021. ABECMA (idecabtagene vicleucel) is approved for the treatment of adult patients with relapsed or refractory multiple myeloma after four or more prior lines of therapy including an immunomodulatory agent and it targets B-cell maturation antigen (BCMA). BCMA is a protein found on the surface of multiple myeloma cells. This was manufactured by Celgene Corporation and approved by the FDA in March 2021. Approval was granted for CARVYKTI (ciltacabtagene autoleucel) recently for the treatment of adult patients with relapsed or refractory multiple myeloma after four or more prior lines of therapy. This is the second B-cell maturation antigen (BCMA)-targeted CAR-T cell therapy. This was manufactured by Janssen Biotech, Inc. and the drug was approved by the FDA in February 2022. Initially, CAR-T cell therapies were developed to target blood cancers, such as myeloma and lymphoma [140,141]. However, with the success observed in these haematological malignancies, researchers have expanded their efforts to develop CAR-T cell therapies for solid tumours as well [142]. A major milestone in this area was reached on February 16, 2024, when the FDA granted accelerated approval to lifileucel (Amtagvi) by Iovance Biotherapeutics, Inc, San Carlos, CA, USA adoptive cell therapy using autologous tumour-infiltrating lymphocytes (TILs), for adult patients with advanced or unresectable melanoma progressing after prior treatment with immune checkpoint inhibitors and BRAF/MEK inhibitors if BRAF V600 is mutation-positive. Amtagvi represents the first individualised TIL therapy to be approved. Similar to CAR-T therapies, it is manufactured using the patient’s immune cells. However, instead of engineering artificial receptors, the therapy harnesses pre-existing TIL cells that can naturally recognise cancer biomarkers. In the single-arm study that supported its approval, Amtagvi shrank tumours in 31.5% of patients previously treated with a PD-1 inhibitor, with 43.5% of responders remaining in remission for over a year. This approval is a significant step forward, bringing adoptive cell therapy approaches to the solid tumour setting. This has resulted in increased investments in research and development, with numerous clinical trials underway to explore their effectiveness in various cancer types [143,144,145]. While initial CAR-T cell therapies targeted antigens like CD19 for blood cancers, the limited presence of tumour-specific antigens, challenges with T cell infiltration, and the immunosuppressive tumour microenvironment have hindered progress for solid tumours [146,147,148]. However, clinical trials are actively assessing CAR-T cell therapies targeting antigens like carcinoembryonic antigen (CEA) across various solid tumour types including gastric cancer, colon cancer, pancreas cancer, oesophagus cancer, lung cancer, and CD70-targeted CAR-T in the treatment of solid tumours. such as renal cell carcinoma, ovarian cancer, and cervical cancer. The approval of Amtagvi validates the tumour-infiltrating lymphocyte approach and provides hope that overcoming the challenges in the solid-tumour setting is achievable. The intersection of research and clinical trials offers hope for addressing the challenges associated with CAR-T cell therapy in treating solid tumours. The dynamic landscape of cancer immunotherapy is constantly evolving, with ongoing efforts to fully unleash the potential of CAR-T cells as a potent weapon against cancer. This personalised immunotherapy approach harnesses the specificity of antibody recognition and the cytotoxic potential of T cells, offering a promising treatment modality for cancers.

### 7.2. Engineered Natural Killer (NK) Cells

The field of cancer treatment has seen a breakthrough in the development of CAR-T cells. Numerous studies conducted in the preclinical stage have demonstrated the exceptional effectiveness of CAR-NK cells in treating cancer [149]. NK cells are specialised white blood cells that play a crucial role in defence against malignant cells in the body. CAR-NK cells offer multiple benefits compared with CAR-T cells. A key advantage lies in their independence from autologous sources, which makes them more readily available for treatment [150]. Additionally, CAR-NK cells have a brief lifetime lowering the likelihood of adverse effects. [151]. Despite these benefits, clinical outcomes of CAR-NK cell treatment have not been as promising as predicted. Low antitumour effects and proliferative potential have been recognised as important drawbacks to the effectiveness of CAR-NK cell therapy [152]. cytokine-induced killer (CIK) cells also have shown great potential in emerging immunotherapy research. CIK cells refer to NK cells that are grown outside of the body and are stimulated with cytokines like interleukin-2 (IL-2) to enhance their anti-tumour activity and cytokine production. Recent research on CIK cells has focused on various aspects, including their role in cancer immunotherapy, their efficacy in combination with other treatments, and their potential as a targeted therapy for different types of cancer. Studies have explored the characteristics of CIK cells, their cytotoxic effects, and their interaction with other immune cells [43,153,154,155]. Research on CIK cells is currently exploring the way they work in conjunction with other therapies, including chemotherapy and checkpoint inhibitors, to improve treatment outcomes. [156,157]. Another area is focused on developing efficient and standardised methods for CIK cell expansion and quality control of CIK cells [43,158,159]. Despite the promising advancements in CIK cell therapy, there are still several challenges that need to be addressed. One of the main challenges is the manufacturing complexity of these cells. Scaling up production while maintaining quality and affordability demands further optimisation. Additionally, long-term safety profiles and potential side effects need careful monitoring in clinical trials. The strategies to enhance in vivo persistence and improve the targeting of solid tumours are crucial to optimise the efficacy of CIK cell therapy.

## 8. Advances in Gene Therapy

Recent advances in gene therapy for cancer have demonstrated a diverse array of strategies, including advanced technologies, such as nanotechnology and CRISPR-Cas9.

### 8.1. Integration of Nanotechnology

The field of nanotechnology has brought remarkable advancements in cancer gene therapy. Gene therapy is a potent treatment method that includes inserting healthy genes into cancerous cells to correct genetic abnormalities [160]. However, a significant hindrance in gene therapy is effectively transporting therapeutic genes to target cells while avoiding adverse reactions [161]. This is where nanocarriers for gene delivery come into play. Nanocarriers are nanoparticles that can encapsulate therapeutic genes and deliver them directly to cancer cells [162]. Nanocarriers have several advantages over conventional gene delivery methods. They can pass through the natural barriers of the body, such as the blood–brain barrier more effectively to reach cancer cells [163]. They can protect therapeutic genes from degradation by enzymes in the body, ensuring that they reach their intended targets intact [164,165]. Moreover, they can be designed to release their payload specifically into cancer cells, minimising off-target effects and reducing toxicity [43,166]. Researchers are focusing on several nanoparticles that have shown promise in gene therapy studies. Apart from viral vectors, such as adeno-associated viruses (AAVs), adenoviruses (AVs), and lentiviruses, significant attention has been directed to non-viral vectors including, lipid nanoparticles (LNPs), polymeric nanoparticles, gold nanoparticles, and carbon nanotubes, and are currently hot topics in gene therapy research.

Lipid nanoparticles (LNPs) have been widely explored in gene therapy studies for gene delivery. Even though many drug candidates, such as TKM-080301, Atu027, DOTAP-Chol-fus1, and DCR-MYC, consisting of LNP formulations entered clinical trials in the past decade, and the majority of them have been unsuccessful as effective gene therapies for cancer treatment [167]. However, despite the drawbacks, researchers are actively engaged in the development of novel formulations. For instance, recent studies registered in clinical trials have shown effective gene delivery of lipid nanoparticles (LNPs) in phase I/II trials, and some LNPs have been successfully employed in delivering DNA plasmid engineered with the TUSC2 tumour suppressor gene in the treatment of non-small-cell lung cancer. In another study, LNPs have been used to enclose mRNAs containing the genes for human IL-23, OX40L, IL-23, and IL-36γ in the treatment of solid tumours, such as triple-negative breast cancer, urothelial cancer, and immune checkpoint refractory melanoma. Furthermore, engineered liposomes have been used in small interfering RNA (siRNA) delivery of Phase I clinical trials in treating solid neoplasm.

Another type of nanocarrier that shows promise in gene delivery is polymeric nanoparticles. While still in the early stages of development, researchers are investigating natural and synthetic polymer-based nanocarriers for effective drug delivery [168,169,170,171], and they have been successful in delivering genetic material to cancer cells in vitro and in animal models, leading to significant reductions in tumour growth [169,172]. However, further studies need to be conducted to assess the safety and efficacy of polymeric nanoparticles in humans. Dendrimers are another type of nanocarrier that show promise in gene therapy. These are highly branched molecules that can carry multiple copies of therapeutic genes [173]. Several studies have demonstrated the potential of dendrimers as gene delivery vectors for cancer treatment. For example, dendrimers have been engineered widely to deliver small interfering RNA (siRNA) to cancer cells. This method has been shown to effectively inhibit the growth of tumour cells in vitro and in vivo [172,174,175].

AuNPs have unique physicochemical properties that offer efficient nucleic acid binding properties. One of the most notable properties of AuNPs is their ability to be coated with cationic molecules, which can alter their surface charge [176]. This alteration improves DNA binding via electrostatic interaction, making them effective carriers of DNA in gene therapy and gene delivery. In addition to this, AuNPs possess the ability to mimic synthetic antibodies due to their simple surface chemistry. This allows for precise control over their binding interactions [176]. The use of AuNPs in cancer gene therapy has been explored in several studies, demonstrating their potential for effective targeted delivery of therapeutic genes. PEGylated AuNP has been studied for its tumour-penetrating efficiency, and researchers have experienced a substantial decrease in the tumour growth rate in mice [177]. Mitra et al. [178] studied an epithelial cell adhesion molecule (EpCAM), which was a monoclonal antibody conjugate of AuNPs loaded with EpCAM-specific siRNA molecules for treating retinoblastoma (RB), and observed a significant downregulation of EpCAM gene. Furthermore, scientists have developed several synergistic gene-delivery systems and conjugates with different gold nanoparticles. Chitosan–gold nanorods were developed for siRNA delivery and showed promising results in gene silencing in triple-negative breast cancer cells [179]. Researchers developed a Au-DENPs-PEG-FA gene delivery system, which was composed of dendrimer–gold nanoparticles engineered with folate–PEGylated conjugate, and it showed low cytotoxicity, good cytocompatibility, and a higher gene transfection efficiency in HeLa cells compared with other vectors [180]. Rattle-structured rough nanocapsules (Au@HSN-PGEA, AHPs) with gold nanorod cores have been studied for co-delivery of antioncogene p53 and sorafenib for treating malignant hepatocellular carcinoma. The study proposed an acceptable strategy for multifunctional nanoparticles with high therapeutic efficacy [181]. Another system, HA/anti-miR-21/PPAuNCs, was developed by Wang et al. [182], condensing microRNA-21 inhibitor into hyaluronic acid (HA) and combining it with polyethylenimine (PEI), and PEGylated gold nanocages (AuNCs). The system has also demonstrated good stability and biocompatibility with precise targeting capabilities. Moreover, Lihuang et al. [183] has revealed that D/R@Ang2-Lip + Au can successfully penetrate the blood–brain barrier and target glioblastoma cells, both in vitro and in vivo, and it has also demonstrated remarkable anti-proliferative effects.

Carbon nanotubes (CNTs) have gained significant attention in gene delivery due to their unique properties, such as their biocompatibility, increased surface area, easy cell membrane penetration, and multifunctional surface chemistry [184]. Furthermore, their ability to efficiently deliver biomolecules into cells, protection of DNA/RNA strands against enzymes, and potential for triggered cargo release make CNTs valuable tools in gene delivery [185]. Researchers have developed single-walled carbon nanotubes (SWCNTs) and multi-walled carbon nanotubes (MWCNTs) with various chemical groups to improve solubility, biocompatibility, targeting ability, and release mechanisms for the genetic material [186,187,188,189]. CNTs have been explored as carriers for the delivery of siRNA. Functionalised CNTs have been demonstrated to be effective in the delivery of siRNA and effective therapeutic silencing in tumour cells, including lung cancer cells and prostate cancer cells [190,191,192,193]. CNTs have also been investigated as carriers for plasmid DNA. A new RNA-wrapped SWCNT delivery system was developed for the delivery of chloroquine and plasmid DNA by Sanz et al. [194], and the system showed efficient gene transfection into HeLa cells and efficient gene expression. Functionalised CNTs hold immense promise for plasmid DNA delivery. In a study conjugating polyethylenimine (PEI) with SWNTs, the highest transfection efficiency in murine neuroblastoma cells was reported in low-molecular-weight PEI conjugates [187]. Kaboudin et al. [195] explored the magnetic functionalisation of MWCNTs using pyridine. They observed easy binding of MWCNTs to cargoes and low cytotoxicity in transferring into the cells.

In addition to these advances, researchers have made progress in the field of nanotechnology by developing theragnostic nanoparticles. These nanoparticles combine diagnostics and therapeutics into a single approach, holding exciting promise for personalised treatments. For instance, Ünal et al. [196] studied iron oxide nanoparticles conjugated with Argonaute 2 protein as a delivery agent, and these nanoparticles effectively delivered microRNA to HER2-positive breast cancer cells. Moreover, when combined with chemotherapy, these nanoparticles enhanced the effectiveness of the treatment both in vitro and in vivo.

The utilisation of nanoparticles in gene therapy for cancer has made significant steps in offering unique capabilities and promising opportunities for personalised and targeted cancer treatment. Recent advancements have overcome initial obstacles, resulting in developments in multifunctional designs and controlled release mechanisms. Despite the potential benefits, there are also concerns about biocompatibility and long-term safety. Therefore, optimising nanoparticle design and minimising potential toxicity remains crucial for clinical translation. However, the integration of nanotechnology in cancer treatment has significantly advanced the field of gene therapy, mainly in targeted drug delivery systems.

### 8.2. CRISPR-Cas9 Genome Editing

**Figure 6:**
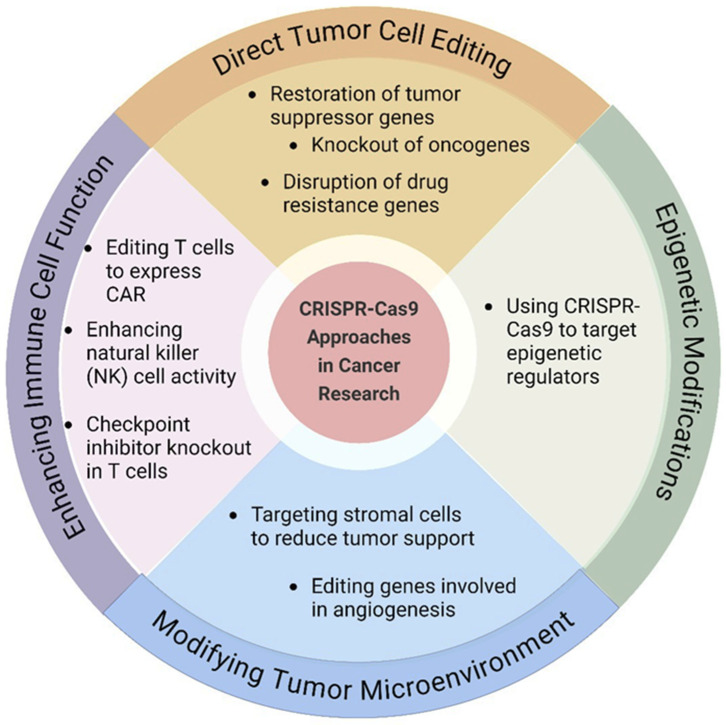
This figure illustrates the key CRISPR-Cas9 gene editing strategies employed in cancer research. These approaches are aimed at either modifying cancer cells or enhancing anti-tumour immune responses. This diagram outlines core focus areas where CRISPR-Cas9 technology is being applied to current research; direct tumour cell editing, enhancing immune cell function, modifying tumour microenvironment, and epigenetic modifications. Each strategy offers unique potential for advancing cancer therapies, highlighting the multifaceted applications of gene editing in oncology.

With the advent of gene editing technologies like CRISPR-Cas9, the field of targeted cancer therapy has created a multitude of previously unimagined possibilities in cancer therapy. CRISPR stands for Clustered Regularly Interspaced Short Palindromic Repeats and it is a natural technique belonging to bacteria for protecting themselves against viruses [197]. Scientists have adapted this system into a potent technique for the precise editing of genes in living cells, and they are working on developing more targeted and personalised treatments for cancer [198]. The CRISPR-Cas9 system consists of two main components, a guide RNA (gRNA) molecule, which is responsible for directing the CRISPR system to a precise location on the DNA, and the Cas9 enzyme, which executes the DNA cleavage at the targeted site [199]. Scientists are currently exploring various CRISPR-Cas9 approaches for gene editing in tumours. Figure 6 depicts the main strategies currently being explored in the field. One approach involves correcting mutations in tumour suppressor genes, which can stop tumour growth by restoring their proper function. For instance, recent studies have shown that CRISPR/Cas9 technology has the potential to target the TP53 tumour suppressor gene, which is frequently mutated in various cancers. By correcting these mutations, researchers expect to improve cancer treatment outcomes and reduce the risk of reappearance [200]. Another strategy focuses on disrupting oncogenes or genes crucial to specific cancer pathways intentionally, which hinders tumour progression and compromises cancer cell survival. Rahimi et al. [201] knocked out the Lipocalin 2 (Lcn2) gene in a prostate cancer cell line (PC3) using CRISPR/Cas9 technology. Lcn2 was reported as a chemo resistant gene and upregulated in many types of cancers. The study reported that gene knockout resulted in reduced cell proliferation, increased sensitivity to chemotherapy and enhanced cell death. Moreover, recently, researchers have investigated the effects of gene knockout of hepatocyte growth factor (HGF) using the CRISPR/Cas9 system in hepatocellular carcinoma (HCC) cells, HCC Huh7 and Hep3B. They observed that transfection of Crispr-HGF resulted in reduced HGF mRNA expression and induced cell death [202]. One of the promising areas of research where the CRISPR-Cas9 technology has been used is tumour angiogenesis.

**Figure 7:**
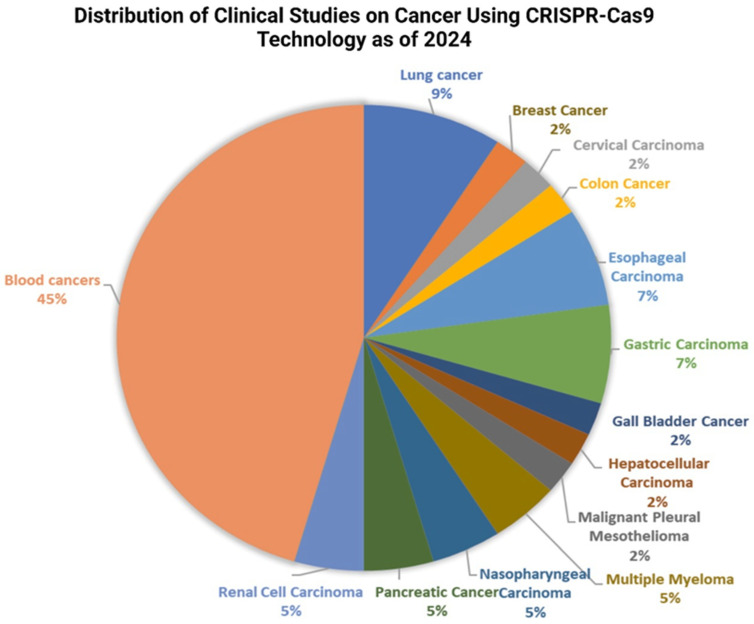
This chart illustrates the current landscape of Phase I/II clinical trials investigating the therapeutic potential of CRISPR-Cas9 technology in cancer treatment. Blood cancers dominate clinical studies, occupying a substantially larger proportion of the studies compared with solid tumours. This highlights the current focus of CRISPR-Cas9 cancer research, with blood cancers receiving considerably more attention in clinical trials than solid tumours.

The system has been used to target and modify the genes involved in tumour angiogenesis. Tumour angiogenesis is the process of the development of new blood vessels that source nutrients and oxygen to the growing tumour and remove waste produced by the tumour cells [203]. This process is crucial for tumour development and metastasis, and is regulated by several genes. Several researches have demonstrated the effectiveness of the CRISPR-Cas9 system in inhibiting tumour angiogenesis. For instance, researchers have used the CRISPR-Cas9 system to target the vascular endothelial growth factor (VEGF) gene, which encodes for a protein that promotes the formation of new blood vessels. Yiu et al. [204] and Ameri et al. [205] used CRISPR-Cas9 technology to disrupt the VEGF-A gene in human retinal pigment epithelial cells, which resulted in a decrease in VEGF-A secretion and a reduction in angiogenesis [204]. Another gene that has been targeted using the CRISPR-Cas9 system is the Hypoxia-inducible factor 1- subunit alpha (HIF-1α) gene, which is involved in the regulation of oxygen levels in cells. A study was conducted on liver cancer cells to investigate the effects of HIF-1α knockout using a lentivirus-intermediated CRISPR/Cas system with small guide RNA-721 (LV-H721), and the results showed that knocking down HIF-1α reduced cancer cell spreading, migration, and cell death under hypoxic conditions [206]. In another study, researchers developed a lipid-based delivery system for CRISPR/Cas9 that targets and suppresses HIF-1α in pancreatic cancer cells. The system successfully inhibited HIF-1α expression, leading to enhanced antimetastatic effects and prolonged survival time in animal models [207]. Moreover, An et al. [208] developed a hypoxia-dependent CRISPR-Cas9 system for the first time by combining the oxygen-sensing transcription activation domain (TAD) of HIF-1α with the gene cleavage and activation ability of Cas9 HEK293 cells. This system has shown promise in the successful modulation of target gene expression under hypoxic conditions by downregulating cancer metastasis-related genes and upregulating HNF4 and NEUROD1 genes. Researchers are implementing regulatory mechanisms using CRISPR technology to modulate gene expression associated with the immune response, and it has been successfully used in editing genes involved in T cell activation and differentiation, enhancing their anti-tumour activity [209,210]. Furthermore, CRISPR-Cas9 technology has emerged as a powerful tool in crafting the next generation of CAR-T cells. Researchers have engineered CAR-T cells with universal characteristics, demonstrating resistance to PD-1 inhibition. These genetically modified CAR-T cells exhibited heightened efficiency in brain tumour models, leading to extended survival in intracranial tumours in mice [211]. In a recent Phase I study, researchers used CRISPR/Cas9 to create PD-1 and T cell receptor (TCR) deficient mesothelin-specific CAR-T cells for treating solid tumours. This approach has shown promising results, with patients experiencing significant tumour shrinkage [212]. Another recent clinical trial used CRISPR/Cas9 gene editing to create universal CAR-T cells that can target both CD19 and CD22, two proteins commonly found on the surface of leukaemia cells. The study discovered that these cells had a regulated safety profile and demonstrated promising antileukemia effects in patients with relapsed or refractory acute lymphoblastic leukaemia [213]. According to the recent data on Clinicaltrials.gov, a growing number of trials are investigating the therapeutic potential of CRISPR-Cas9 technology in cancer treatment. Notably, according to Figure 7, the majority of these trials focus on haematological malignancies, particularly leukaemia and lymphoma, reflecting the established success of CAR-T cell therapy in these malignancies. However, studies targeting solid tumours, such as breast, colorectal, and melanoma, are also emerging. At present, the majority of trials are investigating modifying T cells with CARs specific to tumour antigens, and several studies are focused on directly editing genes within the tumour microenvironment. CAR-T cell engineering aims to enhance T cell potency and overcome tumour immune evasion mechanisms by disrupting immune checkpoint proteins like PD-1 and CD19, while direct tumour editing seeks to disrupt oncogenic pathways or introduce tumour-suppressing genes. Additionally, some trials target patient-specific tumour mutations for a more personalised approach. Currently, most trials are in early phases (Phase I or II), primarily focusing on safety and feasibility assessments. While initial results appear promising, the long-term efficacy and potential side effects of CRISPR-based cancer therapies require further investigation. Overall, these clinical trials demonstrate the escalating potential of CRISPR-Cas9 technology to revolutionise cancer treatment. By engineering immune cells and directly targeting tumour genomes, this powerful tool holds the key to developing more effective, personalised, and potentially curative cancer therapies, warranting continued research and development efforts.

## 9. Discussion

Cancer immunotherapy has emerged as a revolutionary approach in oncology, offering new hope for patients with various types of cancer. This review has highlighted the significant progress made across multiple fronts, including targeted antibodies, immune checkpoint inhibitors, cancer vaccines, cytokine therapies, oncolytic viruses, adoptive cell therapies, and gene therapies. The FDA approval of several immunotherapies, particularly in the realm of CAR-T cell therapies and immune checkpoint inhibitors, marks a significant milestone in cancer treatment. The integration of nanotechnology and advanced gene editing techniques like CRISPR-Cas9 has opened new avenues for more precise and personalised cancer treatments. These technologies offer the potential for improved drug delivery, enhanced therapeutic efficacy, and reduced side effects. The development of cancer vaccines, particularly personalised neoantigen vaccines, represents a promising approach to harnessing the patient’s immune system against their specific cancer.

The application of nanotechnology in personalised immunotherapy is not merely theoretical; numerous preclinical and clinical studies have demonstrated its potential. Zuo et al. [214] discuss how nanotechnology can enhance combination immunotherapy by targeting different pathways, thereby improving treatment outcomes. Similarly, Lu et al. [215] focused on the development of nanoparticle systems that allow for precise localisation and co-delivery of immunomodulators, which is crucial for enhancing the efficacy of lung cancer treatments. These findings are echoed by Li [216], who noted that the design of nanoparticles can be tailored to meet personalised treatment demands, although challenges regarding safety and efficacy remain. Moreover, the role of nanotechnology in modulating the tumour immune microenvironment (TIME) is critical for improving immunotherapy efficacy. Xu et al. [217] presented evidence that nanotechnology-mediated therapies can induce immunogenic cell death, promote antigen presentation, and enhance T cell infiltration into tumours, thereby improving the effectiveness of immune checkpoint inhibitors. This aligns with the observations made by Musetti and Huang [218], who highlight the potential of nanoparticle-mediated remodelling of the TIME to overcome barriers to successful immunotherapy. The ability of nanotechnology to address the complexities of the TIME is further supported by [219] who discussed advances in cell membrane-derived biomimetic nanotechnology for cancer immunotherapy. The pharmacokinetic and pharmacodynamic advantages of nanotechnology are also noteworthy. Jin et al. [220] indicated that nanomaterials such as carbon nanotubes and polymeric micelles have shown considerable benefits in drug design, enhancing the delivery of therapeutic agents while minimising systemic toxicity. This is particularly relevant in the context of personalised medicine, where targeted therapies can significantly reduce collateral damage to healthy tissues. The versatility of nanomaterials allows for the engineering of drug delivery systems that can modify biodistribution and tissue uptake, as discussed by Raj et al. [221]. As research continues to advance, nanotechnology-based strategies offer a promising pathway to more effective, personalised, and safer immunotherapies. However, overcoming regulatory and translational challenges will be crucial to fully realising their potential in clinical oncology.

The efficacy of many immunotherapies is limited to certain cancer types, and questions such as treatment resistance, severe side effects, and the complexity of the tumour microenvironment continue to pose obstacles. The translation of successful treatments for haematological malignancies to solid tumours remains a critical area of focus, as evidenced by the recent approval of Lifileucel (Amtagvi) for advanced melanoma. Recent clinical trials underscore the rapid advancements in cancer immunotherapy, with promising results emerging across multiple treatment modalities. Table 4 highlights the current status of the clinical trials in emerging immunotherapeutics. These findings pave the way for further research into optimising combination strategies and expanding immunotherapy applications to a broader range of malignancies. The development of more targeted and less toxic therapies, improved patient selection through biomarker discovery, and strategies to overcome immunosuppression in the tumour microenvironment are crucial areas for future research.

Recent advances have also revealed the potential of natural and engineered immunomodulators in enhancing the efficacy of cancer immunotherapy. Polysaccharides derived from plant seeds (PSPs) have demonstrated promising immunomodulatory effects, such as enhancing immune cell activity, promoting cytokine production, and protecting immune organs. These low-toxicity natural compounds offer a novel avenue for complementing existing immunotherapies by targeting critical immune pathways and mitigating immunosuppressive conditions in the tumour microenvironment. For example, PSPs derived from seeds like Nigella sativa have been shown to modulate key immune signalling pathways, such as TLR4/NF-κB, which are essential for initiating and sustaining effective immune responses [222]. Similarly, advancements in nanotechnology have led to the development of metal–organic frameworks (MOFs) and metal–organic nanomaterials (MONs) as highly effective carriers for immunotherapeutic agents. The tuneable porous structures and surface functionalities of MOFs allow for precise drug delivery and controlled release, minimising side effects and improving therapeutic efficacy. When functionalised with immunoadjuvants, MOFs further enhance immune activation, providing a synergistic approach to tumour treatment [223]. MONs, with their high surface area, adjustable size, and ability to be easily functionalised with drugs or targeting agents, offer efficient drug delivery, improved bioavailability, and the potential for targeted treatment of tumour cells. MON’s high surface area, tuneable size, and ability to be easily functionalised with drugs or targeting agents enable efficient drug delivery, enhanced bioavailability, and the potential for precise targeting of tumour cells [224].

The challenges surrounding the scalability and affordability of immunotherapies are multifaceted and critical to advancing cancer treatment modalities. One of the primary issues is the inherent complexity involved in the manufacturing processes of these therapies. For instance, the production of induced pluripotent stem cell (iPSC)-derived natural killer (NK) cells, which are increasingly being explored for their therapeutic potential, faces significant hurdles. Traditional sources of NK cells, such as peripheral blood mononuclear cells (PBMCs) and cord blood units (CBUs), suffer from donor variability and limited availability, making it difficult to scale up production for clinical applications [225]. Moreover, the financial implications of developing scalable immunotherapies cannot be overlooked. The costs associated with cancer immunotherapy are often prohibitively high, which limits access for many patients. A study highlighted that therapy-triggered autoimmunity and inflammation can complicate treatment, further escalating costs and complicating patient management [226]. As the demand for therapies such as chimeric antigen receptor (CAR) T-cell therapy increases, the need for cost-effective and scalable manufacturing processes becomes even more pressing. The current supply chain for autologous CAR T-cell therapies is fraught with challenges, including the need for robust manufacturing and distribution models that can ensure timely delivery to patients while managing costs effectively [227]. The scalability of immunotherapies is also hindered by the need for personalised approaches. For example, the generation of patient-derived organoids for drug testing and therapy development presents logistical challenges. Although organoids can closely mimic the tumour microenvironment, their limited availability and the time-consuming processes required for their expansion make high-throughput screening impractical [228]. This limitation underscores the need for innovative solutions that can facilitate the rapid and efficient generation of therapeutic cells at scale. In addition to these logistical and financial challenges, the regulatory landscape poses further obstacles to the scalability of immunotherapies. The approval processes for new therapies, particularly those involving genetic modifications such as mRNA vaccines and CAR T-cell therapies, are complex and can be time-consuming. Concerns regarding immunogenicity, manufacturing scalability, and ethical considerations must be addressed to ensure that these therapies can be brought to market in a timely and cost-effective manner [229,230]. The clinical success of immunotherapies is often accompanied by significant toxicity concerns. ICIs can trigger immune-related adverse events (irAEs) due to their role in enhancing immune activity. By blocking inhibitory pathways like PD-1 and CTLA-4, these therapies can lead to the loss of immune tolerance, causing the immune system to attack healthy tissues and resulting in conditions such as colitis, pneumonitis, and hepatitis. This heightened immune response contributes to inflammation and tissue damage across multiple organ systems [231,232,233]. The severity and occurrence of irAEs depend on factors such as the specific immunotherapy used, patient health, and genetic predisposition. Combination therapies, such as nivolumab and ipilimumab, carry a higher risk due to enhanced immune activation [234]. Additionally, irAEs can appear unpredictably, sometimes emerging early in treatment or months later, making clinical management challenging. While gastrointestinal toxicities typically arise within weeks, endocrine-related events like thyroiditis may develop over a longer period [235]

Managing irAEs involves immunosuppressive treatments, primarily corticosteroids, to control inflammation. Early intervention is crucial, as severe toxicities may require discontinuation of immunotherapy. In some cases, the severity of irAEs has been correlated with treatment efficacy, suggesting that patients who experience more pronounced immune-related toxicities may also derive greater therapeutic benefit from immunotherapy [75,236].

In conclusion, while cancer immunotherapy has made remarkable strides, continued research and innovation are necessary to address existing challenges and expand the benefits to a wider range of cancer types and patients. The field is poised for further breakthroughs, with the potential to transform cancer from a life-threatening disease to a manageable chronic condition for many patients. As our understanding of cancer biology and the immune system deepens, and as technologies continue to advance, the future of cancer immunotherapy holds great promise for improving patient outcomes and quality of life.

## Figures and Tables

**Table 1:** FDA-approved monoclonal antibody therapies currently available for cancer treatment.

Monoclonal Antibody (mAb)	mAb Type	Target	Approval Year	Approved Cancers
Rituximab (Rituxan/MabThera)	Chimeric mAb	CD20	1997	B non-Hodgkin’s lymphoma (NHL), Chronic lymphocytic leukaemia (CLL)
Trastuzumab (Herceptin)	Humanised mAb	HER2	1998	Breast cancer, Gastric cancer
Bevacizumab (Avastin)	Humanised mAb	VEGF	2004	Metastatic colorectal cancer, brain tumours, non-small-cell lung cancer (NSCLC), renal cell carcinoma
Cetuximab (Erbitux)	Chimeric mAb	EGFR	2004	Non-small-cell lung cancer (NSCLC), advanced squamous cell carcinoma of the head and neck
Panitumumab (Vectibix)	Fully human mAb	EGFR	2006	Metastatic colorectal cancer
Pertuzumab (Perjeta)	Humanised mAb	HER2	2012	Breast cancer
Ramucirumab (Cyramza)	Fully human mAb	VEGFR-2	2014	Advanced or metastatic gastric cancer or gastro-oesophageal junction adenocarcinoma
Daratumumab (Darzalex)	Humanised mAb	CD38	2015	Multiple myeloma

**Table 2:** FDA-approved checkpoint inhibitors for cancer.

Checkpoint Target	Checkpoint Inhibitor	Approval Year	Approved Cancers
**PD-1**	Pembrolizumab (Keytruda)	2014	Melanoma, non-small-cell lung cancer (NSCLC), head and neck squamous cell carcinoma (HNSCC), urothelial carcinoma Classical Hodgkin lymphoma, microsatellite instability-high (MSI-H) or mismatch repair-deficient solid tumours
	Nivolumab (Opdivo)	2014	Melanoma, non-small cell lung cancer (NSCLC), melanoma renal cell carcinoma, classical Hodgkin lymphoma, urothelial carcinoma, head, and neck squamous cell carcinoma
	Cemiplimab (Libtayo)	2018	Metastatic cutaneous squamous cell carcinoma (SCC) Advanced cutaneous squamous cell carcinoma (cSCC)
**PD-L1**	Atezolizumab (Tecentriq)	2016	Urothelial carcinoma, non-small-cell lung cancer (NSCLC), bladder cancer, hepatocellular carcinoma
	Avelumab (Bavencio)	2017	<etastatic Merkel cell carcinoma (mMCC) Urothelial carcinoma
	Durvalumab (Imfinzi)	2017	Non-small-cell lung cancer (NSCLC), urothelial carcinoma
**CTLA-4**	Ipilimumab (Yervoy)	2011	Metastatic melanoma
	Tremelimumab (Imjudo)	2022	Unresectable hepatocellular carcinoma Metastatic non-small cell lung cancer

**Table 3:** FDA-approved cancer vaccines for clinical application.

Vaccine	Target Antigen	Use	Approval Year	Cancer Type
Hepatitis B	Hepatitis B virus (HBV) surface antigen (HBsAg)	Preventative	1981	Hepatocellular carcinoma caused by chronic HBV infection
Bacillus Calmette-Guerin (BCG)	Non-pathogenic *Mycobaterium bovis*	Therapeutic	1990	High-risk non-muscle-invasive bladder cancer (NMIBC)
Cervarix (discontinued)	L1 protein of Human papilloma virus (HPV) types 16 and 18	Preventative	2009	HPV-associated cervical, oropharyngeal, anal, penile, and vulvovaginal cancers
Sipuleucel-T (Provenge)	Prostate acid phosphatase (PAP) protein	Therapeutic	2010	Castration-resistant prostatic cancer
Gardasil-9	L1 protein of HPV types 6, 11, 16, 18, 31, 33, 45, 52, and 58	Preventative	2014	HPV-associated cervical, oropharyngeal, anal, penile, and vulvovaginal cancers

Reproduced from Grimmett et al. (2022) with permission from SpringerLink.

**Table 4:** Current clinical trials in cancer immunotherapy.

NCT Number	Immunotherapy	Oncological Indication(s)	Phase of Trial	Outcome
**NCT04191135**	KEYLYNK-009	Triple-Negative Breast Neoplasms	Phase II	Maintenance therapy with pembrolizumab plus Olaparib showed comparable outcomes to pembrolizumab plus chemotherapy in patients with locally recurrent inoperable or metastatic TNBC.
**NCT03815942**	ChAdOx1-MVA 5T4 vaccine	Advanced Prostate Cancer/Advanced Metastatic Prostate Cancer	Phase I/II	An excellent safety profile elicited robust T-cell responses both in circulation and within the prostate gland, supporting its evaluation in efficacy trials.
**NCT02229084**	P10s-PADRE	HR+/HER2 Early Stage Breast Cancer	Phase I/II	Found to be safe and immunogenic when administered alongside standard chemotherapy.
**NCT03662815**	iNeo-Vac-P01	Advanced Pancreatic Cancer Patient	Phase I	Feasible and safe, showing promising antitumor efficacy in patients with advanced solid tumours.
**NCT03697707**	DCP-001	Acute Myeloid Leukaemia in Remission	Phase II	Demonstrated potential in treating AML, warranting further investigation.
**NCT02410733**	FixVac (BNT111)	Melanoma	Phase I	Suggest the general utility of non-mutant shared tumour antigens as targets for cancer vaccination.
**NCT01631357**	Autologous cytokine-induced killer (CIK) cell immunotherapy combined with chemotherapy	Non-Small-Cell Lung Cancer/Squamous Cell Carcinoma	Phase II/III	The combination therapy improved chemotherapy efficacy and demonstrated a favourable safety profile.
**NCT02272855**	HF10—a HSV-1 oncolytic immunotherapy	Unresectable or Metastatic melanoma	Phase II	Demonstrated a favorable benefit/risk profile and induced immune-cell infiltration, showing promising antitumor activity.
**NCT03252808**	HF10 in combination with chemotherapy	Unresectable Pancreatic Cancer	Phase I	The safety and optimal dosing regimen of HF10 alongside chemotherapy
**NCT06136910**	Oncorine (H101) combined with Tislelizumab and chemotherapy	Advanced Non-Small-Cell Lung Cancer/Advanced Non-Small Cell Lung Cancer (NSCLC)	Phase I/II	Evaluating the efficacy and safety of the combination therapy.
**NCT01598129**	ONCOS-102 with low-dose cyclophosphamide	Refractory Solid Tumors	Phase I	Aimed to determine the optimal dose, safety, and preliminary efficacy of the combination therapy
**NCT03206073**	Pexa-Vec (JX-594) in combination with tremelimumab and durvalumab	Colorectal Cancer/Metastatic Colorectal cancer	Phase I/II	Investigating the safety and efficacy of the combination therapy in patients refractory to standard therapies.
**NCT01455389**	DOTAP: Chol-TUSC2 (FUS1) Gene Therapy in Combination with Erlotinib	Non-Small-Cell Lung Cancer	Phase I/II	Demonstrated safety and feasibility of systemic gene therapy using LNPs for delivering the TUSC2 tumor suppressor gene, with evidence of gene expression in tumor biopsies
**NCT03739931**	mRNA-2752—a Lipid Nanoparticle Encapsulating mRNAs Encoding Human OX40L, IL-23, and IL-36γ	Triple Negative Breast Cancer, HNSCC, Non-Hodgkins, Urothelial Cancer, Immune Checkpoint Refractory Melanoma, and NSCLC Lymphoma	Phase I	mRNA-2752 was well-tolerated and showed preliminary evidence of antitumor activity, both as monotherapy and in combination with durvalumab
**NCT01591356**	EphA2 siRNA	Solid Tumors	Phase I	The study concluded that the liposomal siRNA formulation was well-tolerated, with potential therapeutic effects.
**NCT00938574**	Atu027—a liposomal siRNA formulation targeting protein kinase N3 (PKN3)	Advanced Solid Tumors	Phase I	The study demonstrated that Atu027 was safe and showed preliminary evidence of anti-tumor activity.
**NCT03289455**	AUTO3-PA1—a CAR T cell treatment targeting CD19 and CD22	B Acute Lymphoblastic Leukaemia/Recurrent Childhood Acute Lymphoblastic Leukaemia/Refractory Childhood Acute Lymphoblastic Leukaemia/B-cell Acute Lymphoblastic Leukaemia	Phase I/II	Preliminary results indicate promising safety and efficacy profiles.
**NCT01747486**	Autologous CART-19 T cells engineered to express anti-CD19 chimeric antigen receptors	Relapsed or Refractory CLL (3rd Line) or SLL	Phase II	Demonstrated safety and potential efficacy in targeting CD19-positive cancers.
